# Acute Vertigo After CrossFit Workout in a Young Woman With Chiari I Malformation

**DOI:** 10.7759/cureus.13482

**Published:** 2021-02-22

**Authors:** Giacomo Rossettini, Filippo Maselli, Cosimo de Filippis, Firas Mourad, Andrea Lovato

**Affiliations:** 1 Neurosciences, Rehabilitation, Ophthalmology, Genetic and Maternal Infantile Sciences (DINOGMI), University of Genova, Savona, ITA; 2 Sovrintendenza Sanitaria Regionale Puglia, Direzione Regionale Puglia INAIL, Bari, ITA; 3 Neuroscience, University of Padova, Audiology unit at Treviso Hospital, University of Padova, Treviso, ITA; 4 Faculty of Medicine and Surgery, Department of Clinical Science and Translation Medicine, University of Roma “Tor Vergata”, Brescia, ITA

**Keywords:** case reports, arnold-chiari malformation, high-intensity interval training, vertigo, exercise

## Abstract

CrossFit workout is associated with injuries mostly located over the spine and upper limb. Chiari I malformation (CIM) is characterized by migration of the cerebellar tonsils below the foramen magnum and this clinical condition has never been described after high-intense training such as CrossFit. A 19-year-old woman presented to the ED with acute vertigo, nausea, vomiting, and horizontal spontaneous nystagmus; symptoms began after an intense workout session. During neuro-otological examination, spontaneous positional left-beating horizontal nystagmus, normal response to bi-thermal caloric stimulations, and unremarkable cervical vestibular evoked myogenic potentials in both ears were observed; pure tone audiometry showed normal hearing. Central vertigo was suspected; therefore, an MRI was done, which identified a CIM. The patient’s symptoms spontaneously improved at short term. The six-month follow-up MRI confirmed no changes. The subject completely stopped any training and remained asymptomatic over a 12-month follow-up. Our case report is the first describing CIM presented with acute vertigo after a high-intensity training.

## Introduction

CrossFit is a growing modality of high-intensity functional training and high-intensity interval training (HIIT); it has also become one of the top three worldwide fitness trends since 2013 [1-3]. The functional movements performed during this training are executed quickly, repetitively, and with little or no time recovery between sets [4]. According to Glassman, the founder of CrossFit, “three most important rules about any fitness program are safety, efficacy, and efficiency”; however, the methodology that drives CrossFit is entirely empirical [5]. Despite positive influences on body composition and physical fitness were recognized, recent literature confirmed an association with potential emergence of a high enhanced injury risk with training programs such as CrossFit [6]. Musculoskeletal injuries are the most common, especially for novice participants, resulting in working days lost, increased costs for medical treatments, and extensive rehabilitation [6]. Therefore, CrossFit workout injuries represent an increasing phenomenon encompassing different disorders such as low back pain and rhabdomyolysis [7-8]. To the best of the authors’ knowledge, our case report is the first in reporting isolated acute vertigo and horizontal nystagmus as pathognomonic manifestation of a Chiari I malformation (CIM) after a CrossFit workout. That is, we reported the case of a young woman, who was at first diagnosed with a vestibular neuritis, with a final CIM diagnosis after MRI scans.

## Case presentation

**Figure 1:**
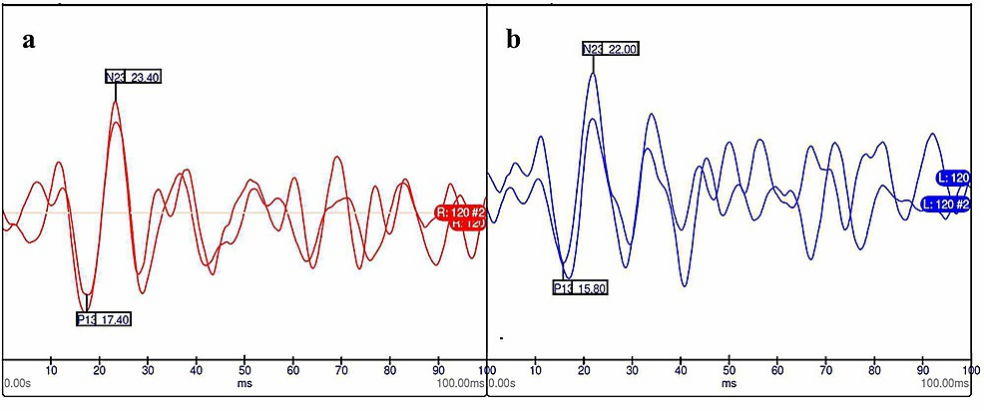
Cervical vestibular evoked myogenic potentials. The patient had normal cervical vestibular evoked myogenic potentials in the right (a) and in the left (b) ear.

A 19-year-old woman self-referred to the ED at Treviso General Hospital complaining of continuous vertigo, nausea, and vomiting that lasted for 48 hours. Her symptoms began after one hour intensive CrossFit training session consisting of power snatch, overhead squat, push jerk, and handstand push up. Vertigo started during the training session forcing the patient to stop. Symptoms rapidly worsened without any reduction of symptoms for two days leading the subject to go to the hospital. Her medical history was unremarkable; fundoscopy was negative; the neurological examinations revealed only postural instability. A horizontal left-beating spontaneous nystagmus was observed during the ear, nose, and throat (ENT) evaluation; also, tilting to the right was observed at the Unterberger test. Accordingly, a diagnosis of vestibular neuritis was done. Then, the subject was admitted to the Internal Medicine Department at Treviso General Hospital where she was treated with IV medication -- betamethasone disodium phosphate 4 mg and metoclopramide monohydrochloride monohydrate 10 mg daily -- with a rapid reduction of the symptom. After three days, an audiological examination was performed and a normal hearing was found [9]. Video-nystagmography showed a left-beating horizontal spontaneous nystagmus; positional nystagmus was horizontal toward the left side without modifications at the head-roll and Dix-Hallpike tests; response at caloric bi-thermal stimulations was normal in both ears. For cervical vestibular evoked myogenic potentials (cVEMPs), a two-independent-channel auditory evoked potential system was adopted (Socrates, Hedera Biomedics, Villanova, Italy). The stimulus was a tone-burst of 500 Hz at an intensity of 120 dB SPL, and it was delivered by earphones. cVEMPs were present, of normal morphology and reproducible bilaterally (Figure 1).

**Figure 2:**
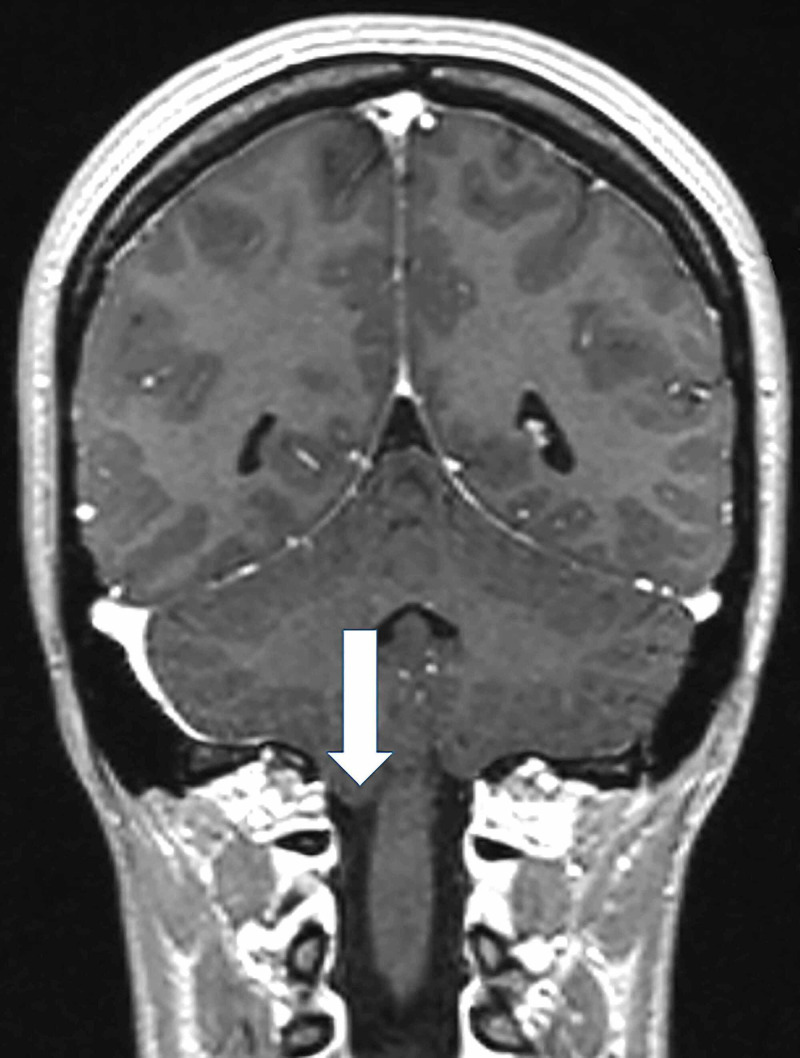
Coronal brain MRI. Coronal brain MRI showing cerebellar tonsil herniation (arrow) through the foramen magnum.

As neuro-otological examination was unremarkable, a central nervous system involvement was suspected. For this reason a contrast-enhanced MRI of the brain was performed. The MRI revealed a cerebellar tonsil herniation through the foramen magnum configuring CIM, without syringomyelia (Figure 2).

The neurosurgeon declared her unfit for surgery due to the symptoms of rapid resolution. The patient was discharged after 10 days of hospitalization. At the three-month follow-up audiological evaluation, the patient was asymptomatic. Video-nystagmography showed a vertical down-beating spontaneous nystagmus and a positional down-beating nystagmus. No changes were observed at six-month follow-up MRI. The subject was advised to repeat a control MRI early. At 12-month follow-up the audiological evaluation and the audio-vestibular examination were still unremarkable. She decided to stop her CrossFit training. The subject provided her consent for this case report.

## Discussion

CrossFit workout is characterized by rapid and high-intensity exercises with marginal recovery session. This type of training reported a high incidence of injuries mostly located over the spine and upper limb [8].

Chiari I malformation is a congenital cranio-cervical malformation characterized by an anatomical defect of the base of the skull in which the cerebellum and the cerebral tonsils herniate through the foramen magnum with no involvement of the brain stem [10]. The pathogenesis of CIM is not clear and includes hydrodynamic and mechanical factors (e.g. physical efforts), hindbrain dysgenesis, and osseous dysplasia [11]. Typically, CIM is discovered in young adults and adolescents with an estimated prevalence between 0.1% and 0.5% [12]. This malformation can be asymptomatic and was discovered incidentally at imaging study [13]. The CIM symptoms are usually occipital and neck pain exacerbated by physical activity or Valsalva maneuvers [14]. A portion of patients presented with symptoms consistent with brainstem or cranial nerve dysfunctions. Patients may develop ataxia, dizziness, nystagmus, sensorineural hearing loss (SNHL), tinnitus, dysphagia, and dysarthria [15].

A recent literature review underlined that unsteadiness (49%) and vertigo (18%) could be developed quite commonly in CIM [16]. Instability may be a part of a cerebellar syndrome caused by the conflict of space in the posterior fossa [14]. Vertigo is considered to be positional or triggered by head movements [12]. Our patients presented with isolated acute vertigo and horizontal nystagmus after a CrossFit workout.

Nystagmus is observed in 15% of CIM patients and it could be horizontal (74%) or down-beating (18%) [16]. Our patients showed horizontal nystagmus at clinical presentation and down-beating nystagmus at follow-up visits when asymptomatic. This change in the direction of the nystagmus could be explained by the pathophysiology of the disease: clinical manifestations of CIM seem to be related to cerebrospinal fluid disturbances and direct compression of nervous tissue that could vary over time [11]. The intense workout could have caused an increase of cerebrospinal fluids and nervous tissue compression, leading to vertigo and horizontal nystagmus.

Our patient had normal response at caloric bi-thermal stimulations and normal cVEMPs in both ears. The findings of a normal labyrinthine function associated with a spontaneous nystagmus, raised the suspicion for central nervous system involvement. A comprehensive neuro-otological examination is mandatory to screen a central vertigo, which could be a potential life-threatening condition [17].

From a differential diagnosis perspective, central vertigo could be the first vestibular attack of a Meniere's disease; however, in this case the symptoms were described as continuous not fluctuating, and the subject did not develop hearing loss during a one-year follow-up [17]. Another possible diagnosis could be a mild unilateral vestibular deficit. According to Ahn et al. these patients typically present with acute isolated vertigo associated with spontaneous nystagmus lasting for longer than one day, but normal response at caloric stimulation [17]. Our patient had a similar presentation but showed persistent signs of central nervous system dysfunction (i.e., the down-beating spontaneous nystagmus).

We report the first case of CIM presented with isolated acute vertigo after CrossFit workout. CrossFit coaches should be conscious about this clinical condition, and exercises should be in line with the training ability of the athletes. Notably, the incidence of delayed diagnosis for serious pathologies of the cervical region is estimated to range from 5% to 20% in the ED: a lack of a misrecognition of them can have life-threatening consequences [18-20].

## Conclusions

This case supports the concept that healthcare professionals are trained and capable of screening for pathologic medical conditions in emergency medicine settings. In conclusion, that is, physician should be aware that a comprehensive neuro-otological examination has to be performed in cases of vertigo with nystagmus in order to identify potential signs of central nervous system lesions.
